# Supplementary material for: Neural Correlates of Numerical Estimation: The Role of Strategy Use
Source: Brain Sci. 2022 Mar 7;12(3):357. doi: 10.3390/brainsci12030357 (PMC8945989; doi:10.3390/brainsci12030357)
Supplement: Supplementary file 1 [file brainsci-12-00357-s001.zip › brainsci-1569857-SI.pdf]

Table S1. Brain areas that showed significant activationin approximate calculation block (thresholding at $p < 0.001$).

Region	Hem.	BA	Number of Voxels	Peak Talairach Coordinates			Peak t(18) Score
				X	Y	Z	
Activations							
Middle frontal gyrus	R	9	20,264	36	35	31	9.734
Insula	R			29	20	8	7.256
Middle frontal gyrus	L	9	16,559	−40	36	26	6.344
Inferior frontal gyrus	L	46		−39	43	8	5.890
Inferior frontal gyrus	L	6		−35	1	25	5.228
Intraparietal sulcus	L	7	21,618	−32	−66	48	8.103
Intraparietal sulcus	L	19		−29	−66	36	7.819
Cuneus	L	7		−9	−72	37	7.665
Intraparietal sulcus	L	40		−50	−44	46	7.931
Cuneus	R	7		8	−76	45	6.320
Intraparietal sulcus	R	7		27	−68	36	5.980
Superior frontal gyrus	L	8	5937	0	11	52	7.700
Insula	L		6366	−18	17	15	6.678
Extra-nuclear/Corpus clausum	L	30	1604	−21	−37	7	6.912
Fusiform gyrus	R	37	44,616	47	−52	−14	8.720
Fusiform gyrus	L	18		−29	−85	−14	6.274
Fusiform gyrus	L	37		−48	−49	−17	6.625
Deactivations							
Middle temporal gyrus	R	22	972	51	−7	−8	6.427
Medial frontal gyrus	L	10	2091	−3	53	13	5.581

Table S2. Brain areas that showed significant activation in sense of magnitude block (thresholding at $p < 0.001$).

Region	Hem.	BA	Number of Voxels	Peak Talairach Coordinates			Peak t(18) Score
				X	Y	Z	
Activation							
Middle frontal gyrus	R	10	23,254	36	44	25	9.689
Middle frontal gyrus	R	6		33	−1	52	6.213
Middle frontal gyrus	L	10	22,212	−36	38	22	9.249
Superior frontal gyrus	L	6	3359	3	2	58	6.580
Supramarginal gyrus	R	40	50,916	30	−52	34	8.639
Inferior parietal lobule	L	40		−42	−46	37	7.289
Inferior parietal lobule	L	7		−34	−64	44	6.529
Posterior cingulate cortex	R	23		6	−10	25	5.439
Insula	R	13	4009	36	11	4	6.207
Inferior occipital gyrus	L	18	67,623	−18	−91	−17	11.845
Inferior temporal gyrus	L	21		−57	−33	−13	7.162
Lateral occipital gyrus	L	37		−39	−62	−14	6.724
Inferior occipital gyrus	R	18		21	−91	−13	7.267
Lateral occipital gyrus	R	37		50	−49	−11	6.331
Deactivation							
None							

Table S3. Brain areas that showed significant differences between approximate calculations compared to sense of magnitude block (thresholding at $p < 0.001$).

Region	Hem.	BA	Number of Voxels	Peak Talairach Coordinates			Peak t(18) Score
				X	Y	Z	
AC strategy > SOM strategy							
Inferior frontal gyrus	R	6	6976	39	−4	35	7.340
Superior frontal gyrus	R	6		0	5	49	9.355
Cingulate gyrus	R	32		8	16	32	6.487
Middle frontal gyrus	R	6		28	−6	52	5.365
Supramarginal gyrus	L	40	12,876	−54	−28	43	9.040
Cuneus	R	7		5	−77	45	11.382
Superior parietal lobule	R	7		24	−73	41	6.113
Inferior parietal lobule	R	7		26	−63	27	5.956
Superior parietal lobule	L	7		−20	−73	44	6.432
Middle occipital gyrus	R	17	1838	24	−88	−11	6.890
Thalamus	L		1064	−7	−5	4	8.260
Thalamus	R			9	−1	1	5.907
Inferior frontal gyrus	L	9	1696	−38	7	26	9.525
Lateral occipitotemporal gyrus	L	37	608	−43	−54	−14	8.870
Inferior occipital gyrus	L	18	2754	−23	−87	−9	8.027
Precentral gyrus	L	6	1061	−30	−7	59	4.023
SOM strategy >AC strategy							
Superior frontal gyrus	R	6	33,350	2	45	37	11.022
Superior frontal gyrus	R	8		21	26	52	11.906
Superior frontal gyrus	L	8		−20	30	51	5.554
Postcentral gyrus	R	4		54	−11	38	5.123
Cingulate gyrus	R	31	170,216	4	−37	40	10.296
Superior occipital gyrus	R	18		14	−76	18	8.654
Middle temporal gyrus	L	21		−44	−7	−15	5.513
Superior temporal gyrus	L	42		−53	−36	12	9.229
Superior occipital gyrus	L	18		−18	−83	16	7.149
Cuneus	L	30		−19	−68	13	7.106
lingual gyrus	R			22	−42	−10	7.447
lingual gyrus	L			−7	−67	−5	6.506
Precuneus	L	31		−13	−37	36	6.791
Inferior temporal gyrus	L	21		−48	−5	−22	7.419
Inferior occipital gyrus	R	37		51	−64	0	6.021
Supramarginal gyrus	R	39		55	−55	22	6.760
Superior temporal gyrus	R	22		48	−32	1	6.752
Middle temporal gyrus	R	21		50	−46	5	5.860
Inferior frontal gyrus	R	46	686	48	31	7	5.528
Inferior frontal gyrus	L	45	3857	−48	38	5	8.285
Inferior frontal gyrus	L	46		−50	31	16	8.412
